# Screening antiproliferative drug for breast cancer from bisbenzylisoquinoline alkaloid tetrandrine and fangchinoline derivatives by targeting BLM helicase

**DOI:** 10.1186/s12885-019-6146-7

**Published:** 2019-10-28

**Authors:** Wangming Zhang, Shuang Yang, Jinhe Liu, Linchun Bao, He Lu, Hong Li, Weidong Pan, Yanchao Jiao, Zhixu He, Jielin Liu

**Affiliations:** 1 The First Affiliated Hospital of Guizhou University of Chinese Medicine, Guiyang, 550001 People’s Republic of China; 2 Department of Immunology, Basic Medical College, Guizhou Medical University, 9 Beijing Road, Guiyang, 550004 People’s Republic of China; 3 Tissue Engineering and Stem Cell Research Center, Guizhou Medical University, Guiyang, 550004 People’s Republic of China; 4 INSERM UMR-S 1165/Paris Diderot 7, Paris, France; 5 INSERM UMR 1234/Faculté de Médecine et de Pharmacie, Université de Rouen, Rouen, France; 6 State Key Laboratory of Functions and Applications of Medicinal Plants, Guizhou Medical University, 3491 Baijin Road, Guiyang, 550014 People’s Republic of China; 7 Guizhou Entry-exit inspection and quarantine bureau, Guiyang, 550004 People’s Republic of China

**Keywords:** BLM helicase, HJNO, Fluorescence polarization, EMSA, MTT, RT-PCR, ELISA

## Abstract

**Background:**

The high expression of BLM (Bloom syndrome) helicase in tumors involves its strong association with cell expansion. Bisbenzylisoquinoline alkaloids own an antitumor property and have developed as candidates for anticancer drugs. This paper aimed to screen potential antiproliferative small molecules from 12 small molecules (the derivatives of bisbenzylisoquinoline alkaloids tetrandrine and fangchinoline) by targeting BLM^642–1290^ helicase. Then we explore the inhibitory mechanism of those small molecules on proliferation of MDA-MB-435 breast cancer cells.

**Methods:**

Fluorescence polarization technique was used to screen small molecules which inhibited the DNA binding and unwinding of BLM^642–1290^ helicase. The effects of positive small molecules on the ATPase and conformation of BLM^642–1290^ helicase were studied by the malachite green-phosphate ammonium molybdate colorimetry and ultraviolet spectral scanning, respectively. The effects of positive small molecules on growth of MDA-MB-435 cells were studied by MTT method, colony formation and cell counting method. The mRNA and protein levels of BLM helicase in the MDA-MB-435 cells after positive small molecule treatments were examined by RT-PCR and ELISA, respectively.

**Results:**

The compound HJNO (a tetrandrine derivative) was screened out which inhibited the DNA binding, unwinding and ATPase of BLM^642–1290^ helicase. That HJNO could bind BLM^642–1290^helicase to change its conformationcontribute to inhibiting the DNA binding, ATPase and DNA unwinding of BLM^642–1290^ helicase. In addition, HJNO showed its inhibiting the growth of MDA-MB-435 cells. The values of IC_50_ after drug treatments for 24 h, 48 h and 72 h were 19.9 μmol/L, 4.1 μmol/L and 10.9 μmol/L, respectively. The mRNA and protein levels of BLM helicase in MDA-MB-435 cells increased after HJNO treatment. Those showed a significant difference (*P* < 0.05) compared with negative control when the concentrations of HJNO were 5 μmol/L and 10 μmol/L, which might contribute to HJNO inhibiting the DNA binding, ATPase and DNA unwinding of BLM helicase.

**Conclusion:**

The small molecule HJNO was screened out by targeting BLM^642–1290^ helicase. And it showed an inhibition on MDA-MB-435 breast cancer cells expansion.

## Background

As one of the biggest public health problems around the world, malignant tumors do great harm to human health and will become the first killer of human in the new century [1]. Conventional cancer treatments such as radiotherapy and chemotherapy cause great damage to normal cells as well as human themselves. Therefore, there is urgent need to develop safer anticancer drugs with fewer side effects.

RecQ helicase family is the most conservative family in the second largest superfamily of helicase. Their members play a pivotal role in keeping genetic stability of various organisms [2], such as DNA replication, repair, recombination, transcription and telomere stability. In humans, there are five kinds of RecQ helicase, those are, RecQ1, BLM, WRN, RecQ4 and RecQ5. The lack of three coding genes BLM, WRN and RecQ4 leads to occur related diseases, which are Bloom syndrome (BS), Werner syndrome (WS) and Rothmund-Thomson syndrome (RTS) [3–5], respectively. The patients of these diseases are commonly susceptible to cancer [6].

BLM helicase is an important member in RecQ helicase family. In human, BLM helicase expressed in various tumors from lymphocytes and epithelial cells. And the expressing BLM in tumors is higher than that in normal tissues [7, 8], implying its strong association with cell proliferation. In esophageal squamous cancer, BLM was reported 2.927 folds increased expression than normal mucosa [9]. Our previous research found the up-regulated expressions of BLM helicase in human leukemia cells and breast cancer cells [10]. Therefore, it provides a new clue to design and screen anticancer drugs by targeting BLM helicase [11–13].

Recently many studies were reported that focused on screening potential anticancer small molecules by inhibiting RecQ helicase. Robert M [14] found that telomycin A and netropsin could inhibit the BLM and WRN helicases. Monika [15] found that NSC 19630 (1-propoxymethyl maleimide) also inhibited WRN helicase. Houqiang Xu [16–20] found that estradiol benzoate and testosterone propionate showed an inhibition on RecQ helicase in *E. coli*, lomefloxacin inhibited DNA unwinding and ATPase of BLM helicase, and Hg^2+^ also inhibited BLM helicase. According to the literatures, bisbenzylisoquinoline alkaloids have an antitumor property and have developed as candidates for anticancer drugs [21]. Tetrandrine and fangchinoline belong to bisbenzylisoquinoline alkaloids. In this paper, potential antiproliferative small molecules for breast cancer were screened out from 12 small molecules (the derivatives of bisbenzylisoquinoline alkaloids tetrandrine and fangchinoline) by targeting BLM helicase. Their inhibiting proliferation were further confirmed by the breast cancer cell growth test. The expressing BLM helicases in breast cancer cells after the small molecule treatments were examined by RT-PCR and ELISA, to explore the small molecule inhibiting cell expansion in breast cancer.

## Methods

### Materials

Recombinant *E. coli* pET-15b-BLM^642–1290^-BL21-CodonPlus was a gift from Dr. Xuguang Xi [22]. MDA-MB-435 breast cancer cells and human umbilical vein endothelial cells HUVECs were gifts from Dr. He Lu [10] and preserved in the Laboratory of Tissue Engineering and Stem Cell of Guizhou Medical University.

### Instruments

AKTA purifier 100 protein separation and purification system (GE Healthcare Co., USA). Beacon 2000 fluorescence polarization analyzer (PanVera LLC, USA). Synergy 4 microplate reader (BioTek Instruments, Inc., USA). SHIMADZU UV-3600 ultraviolet and visible spectrophotometer (Shimadzu Corp., Japan). VCX-500 ultrasonic processor (Sonics & Materials, Inc., USA). Inverted microscope (Nikon Corp., Japan). Gradient thermal cycler (Eppendorf Co., Germany). Milli-Q ultra pure water system (Millipore Corp., USA).

### Chemistry

Twelve derivatives of tetrandrine and fangchinoline such as HJNO were provided by Dr. Weidong Pan’s group. Tetrandrine was selectively halogenated with NXS (X = Cl, Br) in the presence of TFA to obtain compounds HL-5, HL-6, HL-7 and HL-8 [23] and nitrified to obtain compound HJNO [24]. HL-15 was also produced as a major by-product with two nitro groups. The nitro group in HJNO was then efficiently transformed into an amino group by Pd/C in hydrazine hydrate to afford the amino compound, which was added the RCOCl to afford compounds HL-22, HL-24 and HL-27 [23]. HL-25 were synthesized from the amino compound by adding 4-Methylbenzenesulfonyl chloride in pyridine [25]. Fangchinoline reacted with benzoyl chloride in THF in the presence of 4-dimethylaminopyridine (DMAP) to afford HL-23 [26]. Fangchinoline was protected with Bn group, then quaternary ammoniated using BnBr to give HY-2.

HL-15 C_38_H_40_N_4_O_10_ ESI-MS: *m/z* 713.7 [M + H]^+^; ^1^H NMR (CDCl_3_, 400 MHz) *δ* (ppm): 7.42 (1H, s), 7.39 (1H, dd, *J* = 2.4, 8.4 Hz), 7.15 (1H, dd, *J* = 2.8, 8.4 Hz), 6.77 (1H, dd, *J* = 2.4, 8.4 Hz), 6.55 (1H, s), 6.52 (1H, s), 6.30 (1H, dd, *J* = 2.0, 8.4 Hz), 6.00 (1H, s), 3.99 (3H, s), 3.79 (3H, s), 3.71–3.56 (4H, m), 3.44 (3H, s), 3.26 (1H, m), 3.21 (3H, s), 2.96–2.77 (7H, m), 2.66 (3H, s), 2.47 (2H, m), 2.20 (3H, s); ^13^C NMR (CDCl_3_, 100 MHz) *δ* (ppm): 156.7, 151.5, 149.6148.7, 148.5, 144.4, 142.1, 140.7, 138.1, 133.2, 132.6, 129.3, 128.0, 127.7, 127.4, 122.5, 122.3, 121.3, 121.0, 120.6, 120.1, 112.5, 105.9100.6, 64.2, 61.5, 60.0, 56.1, 55.6, 55.5, 45.0, 43.2, 42.3, 40.8, 40.0, 38.7, 24.6, 20.6.

HY-2 C_51_H_53_N_2_O_6_ ESI-MS: *m/z* 790.5 [M + H]^+^; ^1^H NMR (CDCl_3_, 400 MHz) *δ* (ppm): 7.60 (2H, d, *J* = 7.2 Hz), 7.40 (2H, m), 7.17 (5H, m), 7.02 (1H, dd, *J* = 2.4, 8.0 Hz), 6.98 (1H, d, *J* = 8.4 Hz), 6.90 (1H, d, *J* = 2.0 Hz), 6.88 (1H, d, *J* = 4.0 Hz), 6.80 (1H, d, *J* = 8.4 Hz), 6.65 (1H, s), 6.55 (1H, dd, *J* = 2.4, 8.0 Hz), 6.53 (1H, s), 6.48 (1H, dd, *J* = 2.4, 8.0 Hz), 6.33 (1H, d, *J* = 2.0 Hz), 5.68 (1H, s), 5.34 (1H, d, *J* = 12.4 Hz), 4.98 (1H, d, *J* = 10.0 Hz), 4.58 (1H, d, *J* = 10.8 Hz), 4.41 (1H, d, *J* = 10.8 Hz), 3.89 (3H, s), 3.80 (3H, s), 3.76 (1H, d, *J* = 4.8 Hz), 3.50 (3H, m), 3.44 (3H, s), 3.40–2.85 (8H, m), 2.83 (3H, s), 2.76 (2H, m), 2.60 (3H, s); ^13^C NMR (CDCl_3_, 100 MHz) *δ* (ppm): 153.7, 153.0, 149.8, 148.1, 147.8, 146.9, 142.2, 137.5, 136.5, 135.6, 133.2, 133.2, 132.0, 131.1, 130.4, 130.4, 128.8, 128.8, 128.4, 128.4, 128.1, 128.1, 128.0, 128.0, 127.5, 124.3, 123.2, 122.4, 122.3, 119.6, 116.0, 112.7, 112.3, 112.1, 106.1, 74.9, 64.5, 64.2, 64.1, 56.2, 56.1, 55.8, 54.9, 51.1, 45.4, 42.3, 40.5, 40.0, 29.8, 24.9, 24.0.

### Reagents

Positive control mitomycin C (MMC) was from Sigma (USA). 45 nt single stranded DNA (ssDNA, A1: 5′-AATCCGTCGAGCAGAGTTAGGTTAGGTTAGGTTAGTTTTTTTTTT-3′) and fluorescein-labled 21 nt single stranded DNA (ssDNA, A2: 3′-FAM-TTAGGCAGCTCGTCTCAATCC-5′) were synthesized by Beijing Ding Guo Chang sheng Biotechnology Inc. Two complementary ssDNAs were equally mixed in buffer (20 mmol/L Tris, 100 mmol/L NaCl, pH 7.9) and water bath at 85 °C for 5 min. After cooled at room temperature, renaturated double stranded DNA (dsDNA, A1A2) was used as a substrate to detect DNA binding and unwinding of BLM helicase. RPMI-1640 was from Gibco (USA); MTT was from Sigma(USA). Total RNA extraction kit was from Tiangen (China). M-MLV first strand synthesis system reverse transcription kit was from Invitrogen (USA). PCR primers for amplification of *BLM* gene were synthesized by Beijing Ding Guo Biotechnology. The sequence was as follows, forward: 5′-GGATCCTG-GTTCCGTCCGC-3′, reverse: 5′-CCTCAGT-CAAATCTATTTGCTCG-3′.PCR product of *BLM* was 708 bp [27]. β-actin was used as internal control. Its sequence was as follows, forward: 5′-CGGAGTCAA-CGGATTTGGTCGTAT-3′, reverse: 5′-AGCCTTCTC-GATGGTGGTGAAGAC-3′. PCR product of β-actin was 306 bp. Human BLM ELISA kit was from HuaMei Inc. (Wuhan, China). 30% acrylamide and bisacrlamide, TEMED, APS, Glycerol, Tris, bromophenol blue are all from Beijing Solebo Technology Co., Ltd. 5 x TBE buffer was from Beijing Regen Biotechnology Co., Ltd.

### Expression and purification of BLM^642–1290^ helicase

Recombinant *E. coli* pET-15b-BLM^642–1290^-BL21-CodonPlus was seeded into LB media (containing 50 μg/mL Ampicillin + 30 μg/mL Cam) and cultured in a shaker incubator for 190 rpm at 37 °C until OD^600^ reached 0.5–0.6. Expressing BLM helicase was induced by 0.4 mM IPTG for 20 h (18 °C, 190 rpm). After that, bacteria were collected by 4000 rpm centrifuge at 4 °C for 20 min, then ultrasonicated and the supernatant was collected by 13,000 rpm centrifuge at 4 °C for 40 min. The recombinant BLM^642–1290^ helicase used for enzymatic study was harvested after purification by nickel ion affinity chromatography and gel filtration chromatography. Based on the bromophenol blue-stained 10% SDS-PAGE analysis, the purity of the helicase product was above 95%.

### Screening derivatives of tetrandrine and fangchinoline with inhibiting BLM helicase by fluorescence polarization method

We performed fluorescence polarization method to find out the effects of small molecules on the binding between dsDNA and BLM helicase. At first, we added 2 nmol/L fluorescein labeled dsDNA into reaction buffer (20 mmol/L Tris, 25 mmol/L NaCl, 3 mmol/L MgCl_2_, 0.1 mmol/L DTT, pH 7.9) to detect fluorescence anisotropy value in the fluorescence polarization analyzer until it was stable. After that, we added small molecules with different concentrations (0–6.67 μmol/L) and detected fluorescence anisotropy value until it was stable. Finally, 500 nmol/L BLM helicase was added to make DNA substrate soaked and fluorescence anisotropy value was also detected. Total reaction volume was 150 μL by adjusting ddH_2_O volume.

### Detection of the effect of HJNO on DNA binding and unwinding of BLM^642–1290^ helicase determined by fluorescence polarization method

We added 2 nmol/L fluorescence labeled DNA [dsDNA or ssDNA (21 nt)] into reaction buffer (20 mmol/L Tris, 25 mmol/L NaCl, 3 mmol/L MgCl_2_, 0.1 mmol/L DTT, pH 7.9) to detect fluorescence anisotropy value until it was stable. Then we added HJNO with different concentrations (0–33.34 μmol/L) and deccted fluorescence anisotropy value until it was stable. At last 500 nmol/L BLM helicase was added to make DNA substrate soaked and fluorescence anisotropy value was also detected until it was stable. The fluorescence anisotropy values were recorded.

We detected HJNO affecting DNA unwinding of BLM^642–1290^ helicase by using same protocol. The final concentration of HJNO here was 0–50 μmol/L. In addition, in the final step, we added 0.2 mmol/L ATP instead of 500 nmol/L BLM helicase.

### Detection of the effect of HJNO on DNA binding of BLM^642–1290^ helicase determined by EMSA

We added 500 μmol/L fluorescence labeled DNA [dsDNA] into reaction buffer (20 mmol/L Tris, 25 mmol/L NaCl, 3 mmol/L MgCl_2_, 0.1 mmol/L DTT, pH 7.9). Then we added HJNO with different concentrations (0–3.35 μmol/L) and 2.5 μmol/L BLM^*642–1290*^ helicase respectively to make DNA substrate soaked. All reaction tubes incubated for 45 min at room temperature. After 45 min, each tube was added 4 μl loading buffer to end the reaction. We loaded the samples and taken 200v constant voltage electrophoresis for 30 min. Then we observed and recorded the results on the Bio-rad ChemiDoc™ Imaging System.

### The effect of HJNO on the ATPase of BLM^642–1290^ helicase detected by malachite green-phosphate and ammonium molybdate colorimetry

We mixed 125 nmol/L BLM helicase, 100 nmol/L ssDNA (45 nt) and various HJNO solutions (0–100 μmol/L) into reaction buffer respectively. The total reaction volume was 75 μL by adjusting ddH_2_O volume. We incubated the mixture at room temperature for 10 min. Then we added 2 mmol/L ATP into the mixture and incubated it at room temperature for 20 min. Fifty microliter mixture was quickly added into 850 μL dye to terminate the ATP hydrolysis reaction. After 1 min, 100 μL 34% citric acid solution was added to stop color reaction. After that, we added the 100 μL mixture into one well of a 96-well plate and read three repeated wells at the length of 660 nm. The international unit was applied to define the enzyme amount. That is, a unit of enzyme is needed to hydrolyze 1 μmol substrate per minute. The enzymatic amount (units/mL) was calculated as: ${A}_{activity}=\frac{3A}{10B}$.

A was the phosphate concentration (μmol/L) calculated by standard curve. B was reaction time (min).

Relative ATPase activity was equal to the ratio of ATPase activity of BLM helicase treated with HJNO and ATPase activity of that without any treatment.

### The effect of HJNO on the ultraviolet spectrum of BLM^642–1290^ helicase

We mixed 500 nmol/L BLM helicasewith various HJNO solutions (0–50 μmol/L) in Tris-HCl buffer (pH 7.9) respectively. And the total reaction volume was 3000 μL. Then the mixture was scanned by the ultraviolet spectrophotometer at 220–380 nm. The length interval was 0.5 nm and the scanning speed was medium. The scanning interval was 3 min until it was stable. Whether protein conformation changed could be determined by changes of peak shape and position [28, 29]. In addition, we used the same method to scan the ultraviolet absorption spectra of various HJNO solutions (0–50 μmol/L) in the buffer respectively.

### HJNO inhibiting MDA-MB-435 breast cancer cells expansion

#### MTT method

We seeded MDA-MB-435 breast cancer cells into 96-well plate at the density of 8 × 10^3^each well and cultured for 12 h when they were adherent. Then we addedHJNO solutions with different concentrations (0.5 μmol/L, 2.5 μmol/L, 5 μmol/L, 25 μmol/L and 50 μmol/L) respectively. RPMI-1640 complete medium and MMC with the same gradient concentrations as those of HJNO solutions were used as negative control and positive control, respectively. Triplicates were performed. The cells were cultured for 24 h, 48 h and 72 h, respectively. Then we added MTT solution and continued to incubate it for 4 h. After crystalline substance was completely dissolved by DMSO, the automatic microplate reader was used to detect the OD value of each well (wavelength was 490 nm). The inhibition ratio and IC_50_ (50% inhibiting concentration) of drug on the cell expansion were calculated according to the OD values.

#### Cell colony formation

We seeded MDA-MB-435 cells into 24-well plate at the density of 350 each well added of HJNO solutions with different concentrations (0.5 μmol/L, 2.5 μmol/L and 5 μmol/L) respectively. Triplicates were performed. RPMI-1640 complete medium and MMC were used as negative control and positive control, respectively. We had cultured the cells for 7 days. After washed by PBS, cells were fixed by methanol and stained by trypan blue. We calculated the colony forming ratio and colony inhibiting ratio after counting the colonies.

$$ \mathrm{Colony}\ \mathrm{forming}\ \mathrm{ratio}=\left(\mathrm{the}\ \mathrm{number}\ \mathrm{of}\ \mathrm{colonies}/\mathrm{the}\ \mathrm{number}\ \mathrm{of}\ \mathrm{seeded}\ \mathrm{cells}\right)\times 100\% $$

$$ {\displaystyle \begin{array}{l}\mathrm{Colony}\ \mathrm{in}\mathrm{hibiting}\ \mathrm{ratio}=\left(1\hbox{-} \right(\mathrm{colony}\ \mathrm{forming}\ \mathrm{ratio}\mathrm{n}\ \mathrm{in}\ \mathrm{the}\ \mathrm{experimental}\ \mathrm{group}/\mathrm{colony}\ \mathrm{forming}\ \mathrm{ratio}\ \mathrm{in}\ \mathrm{the}\\ {}\mathrm{control}\ \mathrm{group}\left)\right)\times 100\%.\end{array}} $$

#### Cell counting

We cultured and treatedMDA-MB-435 cells as above. After decanting medium, cells were washed by PBS three times and digested by 0.25% trypsin. We added medium with 10% serum to stop the digestion and added 10 μL cell suspension to the cell count plate and then counted the number of cells. The total cell count was calculated by the following equation: Total cell count = N/4 × 104/mL × 0.5 mL (N: the number of cells in the four large squares at the four corners).

### The effect of HJNO on the expression of BLM helicase in the MDA-MB-435 breast cancer cells

The mRNA and protein expression of BLM was detected by RT-PCR and ELISA according to the kit instruction, respectively.

### Statistical analysis

All data are analyzed using SPSS 17.0 statistical software. Compared with BLM expression level in the drug treated group and without drug control group, two independent sample t tests were used to indicate that the difference was statistically significant with *P* < 0.05.

## Results

### Screening out small molecules with inhibiting BLM^642–1290^ helicase from 12 derivatives of tetrandrine and fangchinoline

**Fig. 1:**
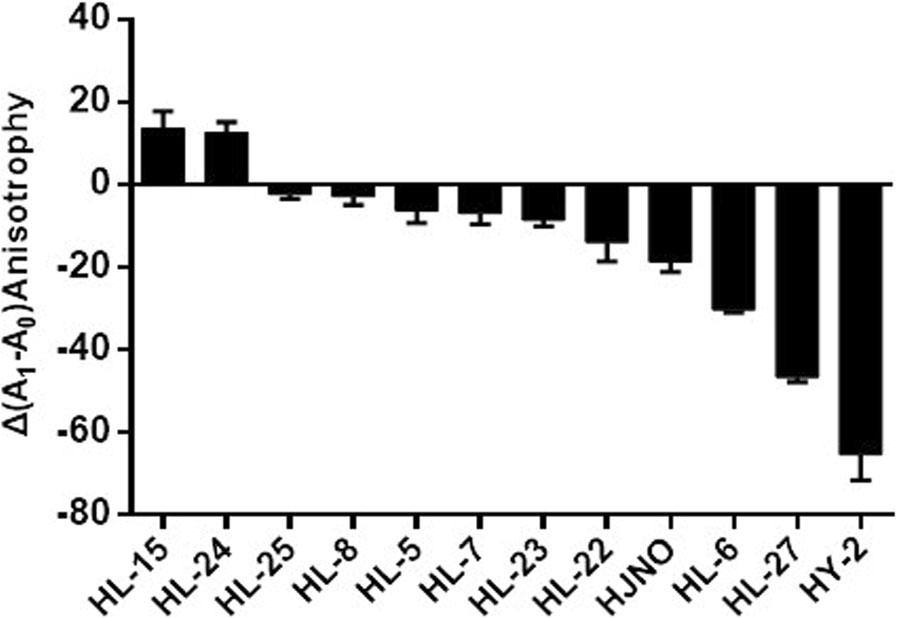
Effects of the derivatives of tetrandrine and fangchinoline on dsDNA binding of BLM^642–1290^ helicase. Note: A0 is the fluorescence anisotrophy of dsDNA binding small molecules. A1 is the fluorescence anisotrophy of the complexes which is formed by BLM^642–1290^ binding dsDNA and small molecules. Δ(A1-A0) is the differences between the activities of BLM helicase binding dsDNA which is treated by small molecules or not. Data were means ± SD with five replicates and the same below

When concentration of small molecules was 6.67 μmol/L, among 12 derivatives of tetrandrine and fangchinoline, the inhibiting values of HL-22, HJNO, HL-6, HL-27 and HY-2 on BLM^*642–1290*^ helicase binding to dsDNA were 14, 19, 30, 47 and 65, respectively (Fig. 1). According to results showed in Fig. 1, we slected and used Tetrandrine HJNO for following experiments.

### The effect of HJNO on the DNA binding of BLM^642–1290^ helicase

**Fig. 2:**
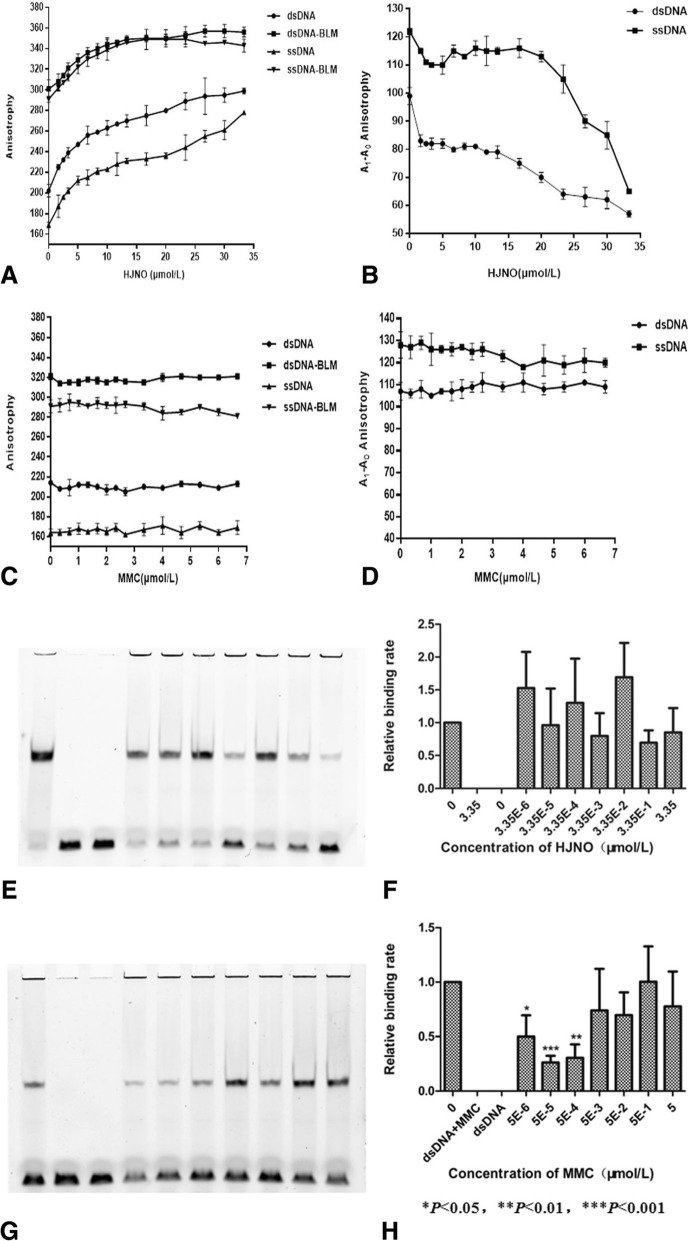
Effects of HJNO and MMC on the DNA binding of BLM^642–1290^ helicase. **a** Effects of HJNO on fluorescence anisotrophy of dsDNA or ssDNA (21 nt) and complexes of BLM^642–1290^ binding dsDNA or ssDNA (21 nt). **b** Effects of HJNO on the dsDNA or ssDNA (21 nt) binding of BLM^642–1290^ helicase. **c** Effects of MMC on fluorescence anisotrophy of dsDNA or ssDNA (21 nt) and complexes of BLM ^642–1290^ binding dsDNA or ssDNA (21 nt). **d** Effects of MMC on the dsDNA or ssDNA (21 nt) binding of BLM ^642–1290^ helicase. **e**, **f** Effects of HJNO on complexes of BLM^642–1290^ binding dsDNA detected by EMSA and statistic results. **g**, **h** Effects of MMC on complexes of BLM ^642–1290^ binding dsDNA detected by EMSA and statistic results. Note: A_0_ is the fluorescence anisotrophy of DNA binding small molecular substances. A_1_ is the fluorescence anisotrophy of complexes which is formed by BLM binding DNA and small molecules

As shown in Fig. 2a, HJNO bound to dsDNA or ssDNA (21 nt) to form a complex. HJNO could inhibit BLM helicase binding to dsDNA or ssDNA (21 nt) and the inhibiting constant (Ki) value was 12.89 ± 3.59 μM or 21.39 ± 1.76 μM (Fig. 2b). When concentration of HJNO was 33.34 μM, the inhibiting ratio of HJNO on BLM helicase binding to dsDNA or ssDNA (21 nt) was 42.42% or 46.72%. While MMC did not bind to dsDNA nor ssDNA (21 nt) (Fig. 2c). MMC had no significant effect on BLM helicase binding to dsDNA and a weak inhibiting effect on BLM helicase binding to ssDNA (21 nt) with the Ki value of 3.62 ± 0.84 μmol/L (Fig. 2d). When concentration of MMC was 6.67 μmol/L, its inhibiting ratio on BLM helicase binding to ssDNA (21 nt) was 8%. As shown in Fig. 2e and f, HJNO suppressed dsDNA binding to BLM^*642–1290*^ helicase at 0.335 μmol/L and 3.35 μmol/L as consistent with the results detected by fluorescence polarization method. MMC had no significant effect on BLM^*642–1290*^ helicase binding to dsDNA detected by EMSA, when concentrations of MMC was 0.5 μmol/L and 5 μmol/L. These results were consistent with the results detected by fluorescence polarization method. But when concentrations of MMC were lower than 0.05 μmol/L, they could inhibit BLM^*642–1290*^ helicase binding to dsDNA. (Fig. 2g and h).

### The effect of HJNO on DNA unwinding of BLM^642–1290^ helicase

**Fig. 3:**
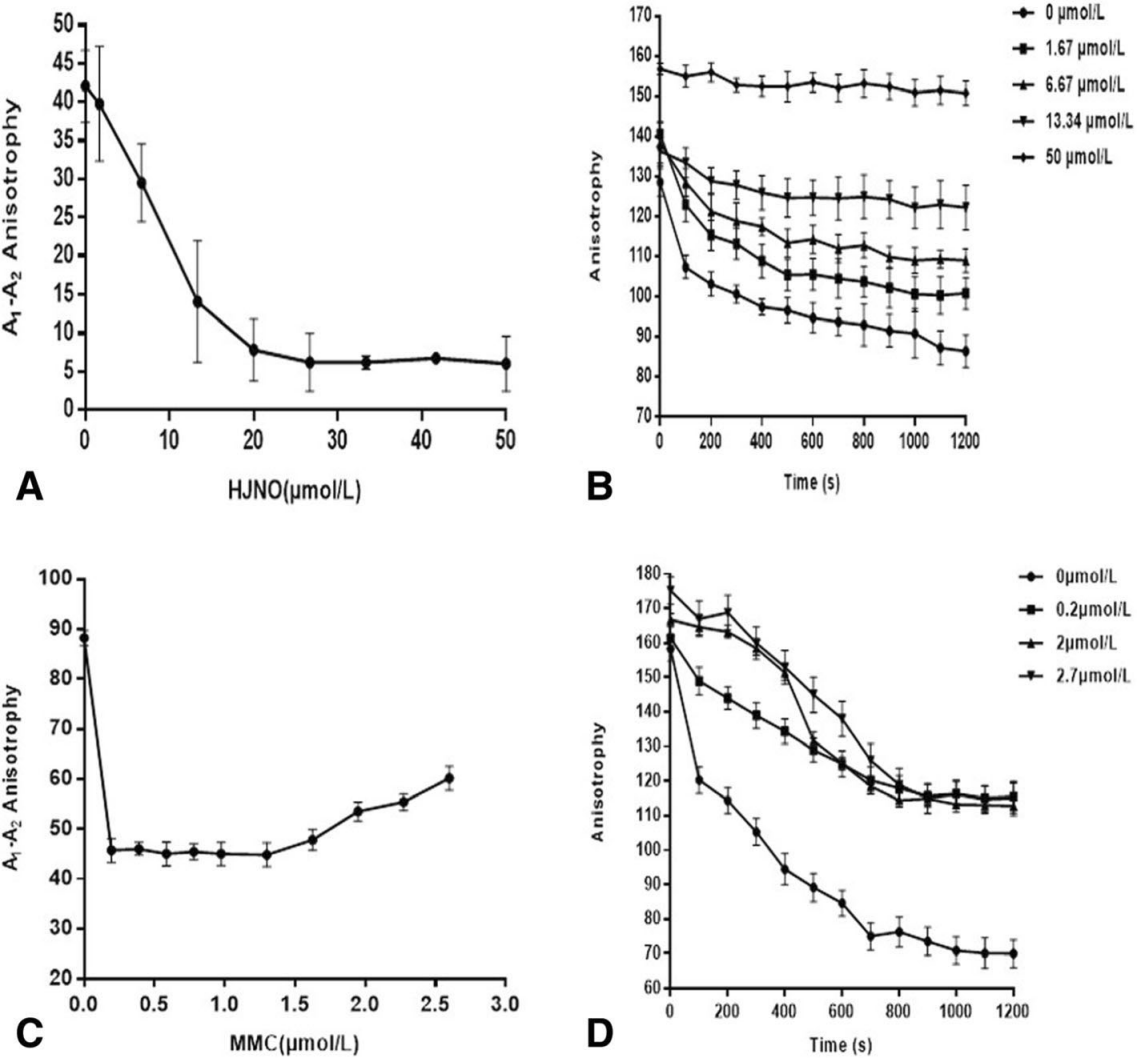
Effects of HJNO or MMC on DNA unwinding of BLM helicase. **a**The effects of HJNO on DNA unwinding of BLM helicase. **b** The effects of 1.67, 6.67, 13.34 and 50 μmol/L HJNO on DNA unwinding time curve of BLM helicase. **c** The effects of MMC on DNA unwinding of BLM helicase; **d** The effects of 0.2, 2 and 2.7 μmol/L MMC on DNA unwinding time curve of BLM helicase. Note: A1 is the fluorescence anisotrophy of BLM binding DNA and small molecules. A2 is the fluorescence anisotrophy after adding 0.2 mmol/L ATP

HJNO could inhibit DNA unwinding of BLM helicase, whose Ki value was 15.62 ± 0.74 μmol/L. When concentration of HJNO was 50 μmol/L, its inhibiting ratio on DNA unwinding of BLM helicase reached 85.74% (Fig. 3a and b). In addition HJNO also exerted an inhibiting effect on DNA unwinding rate of BLM helicase (Fig. 3b).

MMC had a little inhibitory effect on DNA unwinding of BLM helicase as well, whose Ki value was 0.35 ± 0.03 μmol/L (Fig. 3c and d). When concentration of MMC was 1.5 μmol/L, its inhibiting ratio on DNA unwinding of BLM helicase was 49.20%. However, when MMC concentration exceeded 1.5 μmol/L, MMC inhibiting DNA unwinding of BLM helicase decreased.

### The effect of HJNO on the ATPase activity of BLM^642–1290^ helicase

**Fig. 4:**
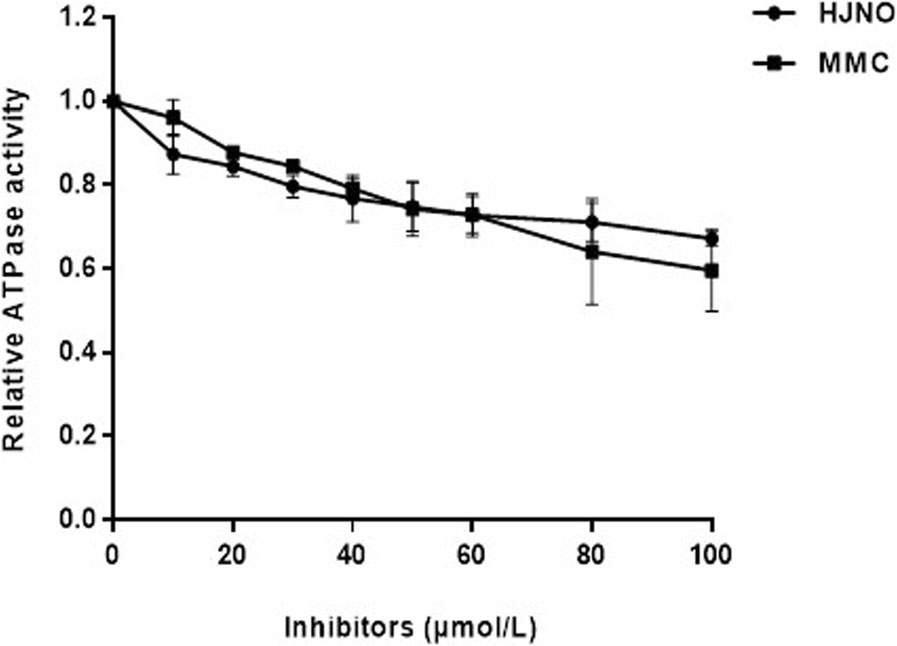
Effects of HJNO or MMC on the ATPase activity of BLM helicase

When concentration of HJNO was 100 μmol/L, its inhibiting ratio on the ATPase activity of BLM helicase was 32.8%, while that of MMC was 40.4% when its concentration was 100 μmol/L (Fig. 4).

### The effect of HJNO on the ultraviolet spectrum of BLM^642–1290^ helicase

**Fig. 5:**
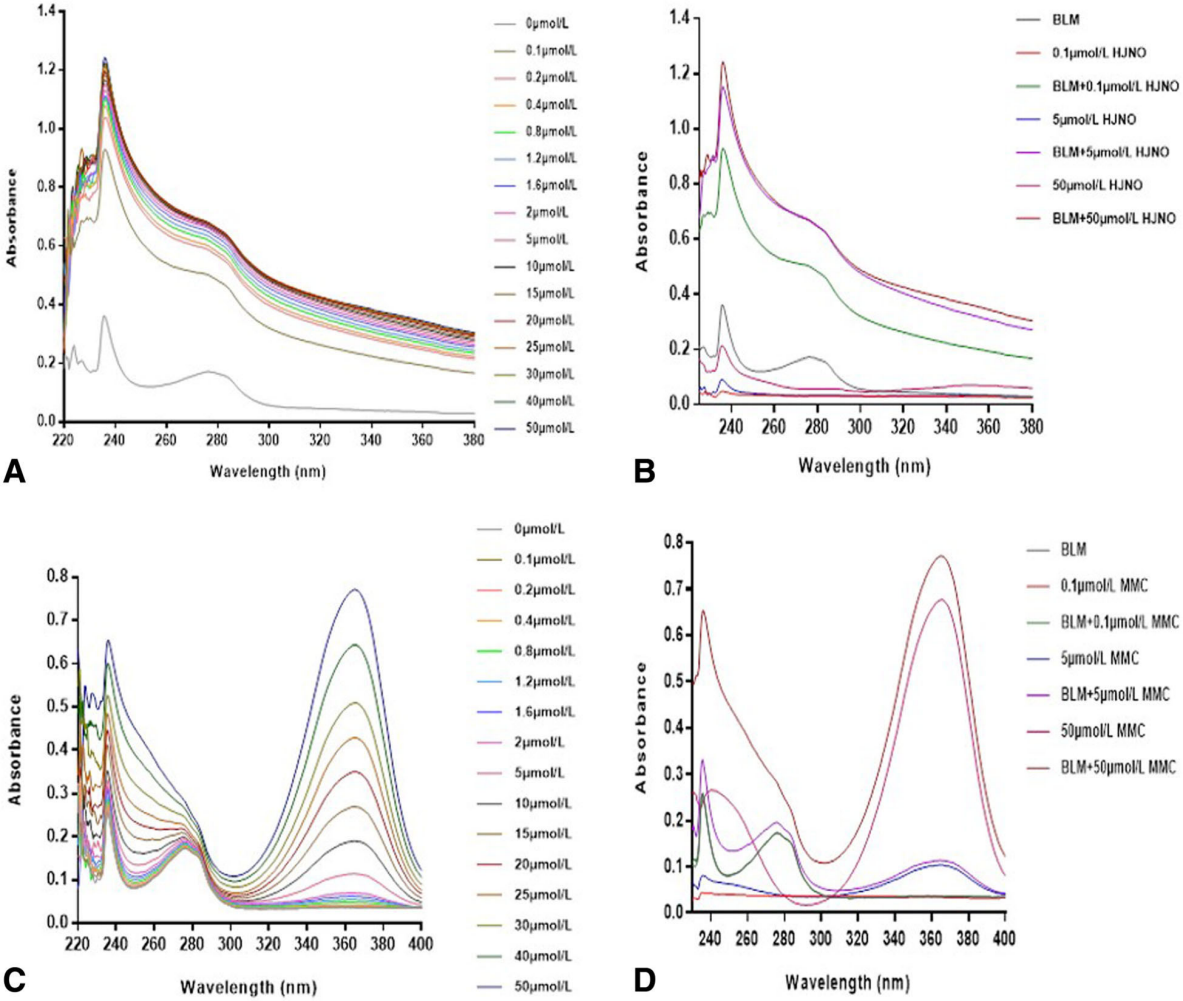
Effects of HJNO and MMC on the ultraviolet aborption of BLM helicase. **a** Effects of different concentration of HJNO on the ultraviolet absorption spectrum of BLM helicase (500 nM). **b** Effects of HJNO (0.1 μmol/L, 5 μmol/L, 50 μmol/L) on the ultraviolet absorption spectrum of BLM helicase (500 nM). **c** Effects of different concentration of MMC on the ultraviolet absorption spectrum of BLM helicase (500 nM). **d** Effects of MMC (0.1 μmol/L, 5 μmol/L, 50 μmol/L) on the ultraviolet absorption spectrum of BLM helicase (500 nM)

As shown in Fig. 5b, the ultraviolet absorption values at 237 nm and 277 nm after HJNO interacting with BLM^*642–1290*^ helicase were more than the sum of those of HJNO and BLM^*642–1290*^ at the same wavelength. It was caused by the chromophore of BLM helicase flipping to a greater polar domain [25]. The ultraviolet absorption values at 237 nm and 277 nm after HJNO interacting with BLM^*642–1290*^ helicase increased with the increasing of HJNO concentration (Fig. 5a). These results suggested that HJNO bound to BLM^*642–1290*^ helicase and changed its conformation.

The ultraviolet absorption values at 237 nm, 277 nm and 365 nm after MMC interacting with BLM^*642–1290*^ helicase nearly equaled to the sum of the ultraviolet absorption values of MMC and BLM^*642–1290*^ helicase at these three wavelengths. It suggested that MMC did not change BLM^*642–1290*^ helicase conformation (Fig. 5d). The ultraviolet absorption values at 237 nm and 365 nm also increased with MMC concentration increasing, while the ultraviolet absorption peak at 277 nm gradually disappeared (Fig. 5c), which was caused by the lack of phenyl group in MMC. The ultraviolet absorption peak still formed by the aromatic residue of BLM^*642–1290*^ helicase, while BLM^*642–1290*^ helicase concentration did not increase. Therefore, MMC exerted no effect on BLM^*642–1290*^ helicase conformation.

### Inhibiting of HJNO on MDA-MB-435 breast cancer cell expansion

**Fig. 6:**
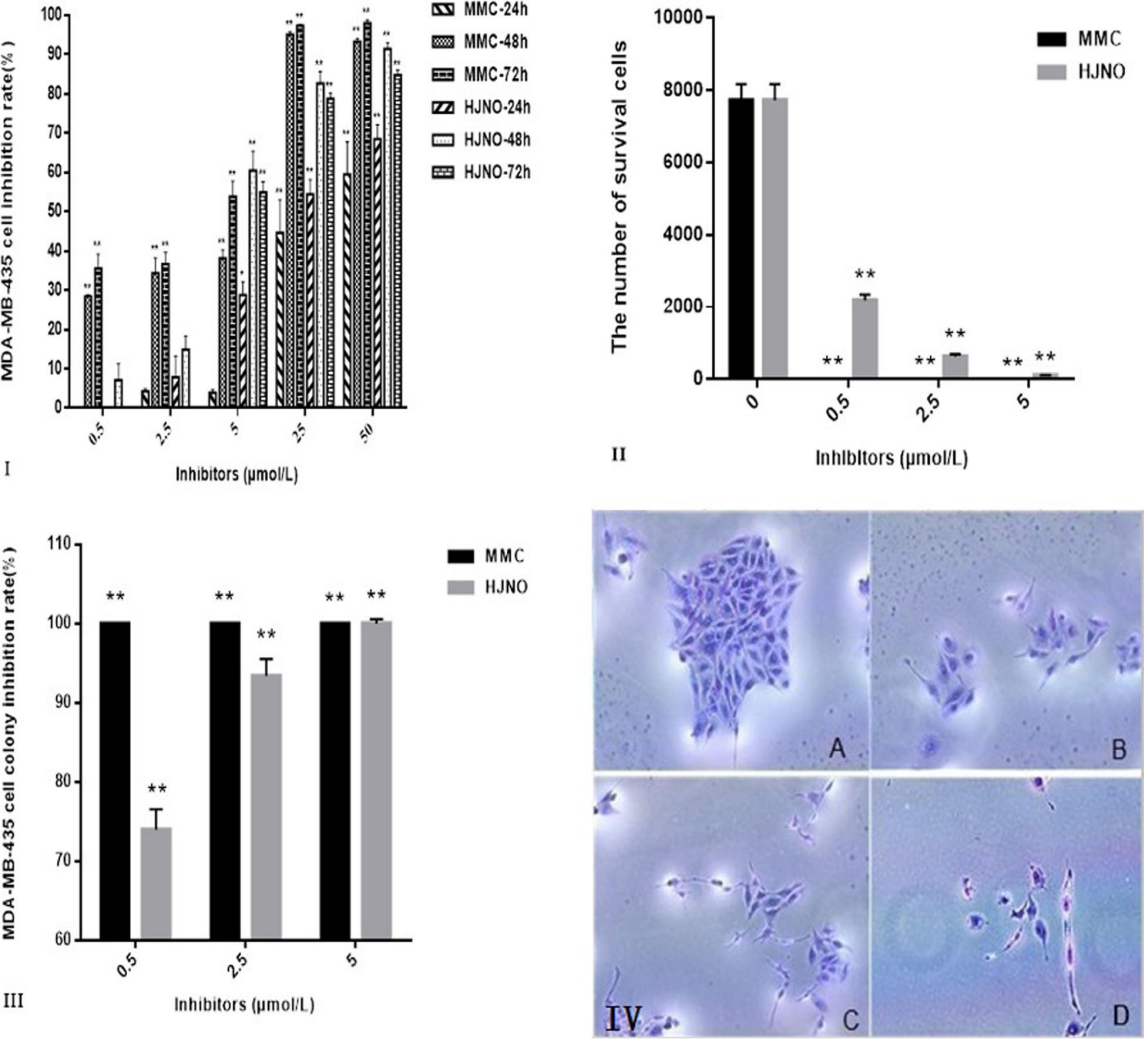
**I** :Effects of HJNO on the growth of MDA-MB-435 cells. **II**: Effects of HJNO on the number of survival MDA-MB-435 cells. **III**, **IV**: Effects of HJNO on the colony formation of MDA-MB-435 cells (400×).Note:A: 0 μmol/L. B: 0.5 μmol/L. C: 2.5 μmol/L. D: 5 μmol/L. “*“*P*<0.05, “**“*P*<0.01, compared with HJNO (0 μmol/L)

The results from MTT test showed that inhibiting ratios of HJNO on expansion of MDA-MB-435 cells increased with HJNO concentration increasing. When HJNO concentrations were 25 μmol/L and 50 μmol/L, its inhibiting ratios for 48 h and 72 h reached around 80% (Fig. 6i). IC_50_ values of HJNO on the MDA-MB-435 cells for 24 h, 48 h and 72 h were 19.9 μmol/L, 4.1 μmol/L and 10.9 μmol/L, respectively, while those of positive control MMC were 30.9 μmol/L, 7 μmol/L and 4.9 μmol/L. The above results showed that inhibiting of HJNO for 24 h and 48 h were stronger than MMC, while MMC exceeded HJNO for 72 h. This suggested that HJNO had a stronger inhibiting on MDA-MB-435 cells expansion in a short time.

Cell counting also showed HJNO inhibiting MDA-MB-435 cells expansion. The inhibiting ratio reached 98.72% when HJNO’s concentration was 5 μmol/L (Fig. 6II).

The results from colony forming assay showed that HJNO inhibited MDA-MB-435 cell forming colonies. When HJNO concentrations were 0.5 μmol/L, 2.5 μmol/L and 5 μmol/L, the colony inhibiting ratios of HJNO on the MDA-MB-435 cells were 74, 93.4 and 100%, respectively (Fig. 6III, IV).

### The effect of HJNO on the expression of BLM helicase in the MDA-MB-435 cell line

**Fig. 7:**
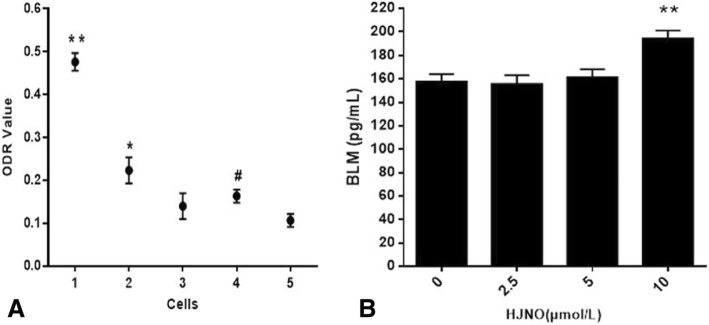
BLM expression in MDA-MB-435 cells induced by HJNO. **a** BLM mRNA expression in MDA-MB-435 cells induced by HJNO (optical density ratio). **b** BLM protein expression in MDA-MB-435 cells induced by HJNO. Note:1: MDA-MB-435 cells (HJNO 10 μmol/L). 2: MDA-MB-435 cells (HJNO 5 μmol/L). 3: MDA-MB-435 cells (HJNO 2.5 μmol/L). 4: MDA-MB-435 cells (HJNO 0 μmol/L). 5: HUVEC cells (HJNO 0 μmol/L). *P < 0.05, **P < 0.01, compared with MDA-MB-435 cells (HJNO 0 μmol/L); ^**#**^*P* < 0.05, compared with HUVEC cells (HJNO 0 μmol/L)

The RT-PCR results (Fig. 7a) showed that BLM helicase mRNA in MDA-MB-435 cells after HJNO treatment for 24 h was significantly higher than that in HUVEC cells. (*P* < 0.05) When the concentrations of HJNO were 5 μmol/L and 10 μmol/L, BLM mRNA was significantly higher than that without HJNO (*P* < 0.05).

The results from ELISA test (Fig. 7b) showed that BLM helicase protein expression increased in MDA-MB-435 cells with HJNO treatment for 24 h. When HJNO concentration reached 10 μmol/L, BLM protein expression was significantly higher than that without HJNO treatment (*P* < 0.01).

## Discussion

Some conservative domains in the RecQ helicases have been identified by sequence analysis. They are unwinding domain (Helicase), RecQ conservative domain (RecQ-Ct) and helicase-ribonuclease D-C terminal domain (HRDC) [30, 31]. HRDC domain is mainly responsible for DNA binding. Helicase domain can not only unwind dsDNA but also show an ATPase activity that binds to ATP and hydrolyze it to release energy. RecQ-Ct domain plays a role in regulating DNA binding and interaction between proteins. Up to now, the common methods for detecting the DNA binding and unwinding of RecQ helicase are fluorescence polarization method, electrophoresis after unwinding the labed DNA and autoradiography. In the present study, we used fluorescence polarization technology, which could intuitively monitored the biological process of BLM ^*642–1290*^binding and unwinding dsDNA. When used in drug screening, It will realtime track and detect the drug-DNA interaction, the effect of drug on the DNA binding of BLM^*642–1290*^ helicase, as well as the effect of drug on the dsDNA unwinding of BLM^*642–1290*^ helicase.

### Tetrandrine derivative HJNO inhibiting DNA unwinding of BLM helicase

Double benzyl isoquinoline alkaloids have anticancer effect and have developed as anticancer drugs [32]. Both tetrandrine and fangchinoline belong to double benzyl isoquinoline alkaloids. Tetrandrine has inhibiting effect on breast cancer [33], prostate cancer cells [34], neuroblastoma TGW [35] and colon cancer cells [36, 37]. Therefore, our study applied BLM^*642–1290*^ helicase inhibiting model and screened out anticancer small molecules from the derivatives of double benzyl isoquinoline alkaloids tetrandrine and fangchinoline. The results showed that we preliminarily screened out five small molecules with inhibiting DNA binding of BLM^*642–1290*^ helicase from 12 derivatives of double benzyl isoquinoline alkaloids. HL-6, HJNO, HL-22 and HL-27 were derivatives of tetrandrine while HY-2 was a fangchinoline derivative.

Anticancer by targeting DNA helicase is that drug interacts with DNA and changes it to interfere DNA helicase. It influences various kinds of cell biological activity such as DNA replication, repair and transcription [11, 38], which is also the primary idea for screening potential anticancer small molecules by BLM^*642–1290*^ helicase inhibiting model. Our study revealed that HJNO could bind to both fluorescence labeled ssDNA and dsDNA, and its binding with ssDNA was stronger than dsDNA. DNA structure was modified by binding with HJNO, thus DNA binding to BLM^*642–1290*^ helicase was inhibited. HJNO inhibiting BLM^*642–1290*^ helicase binding with ssDNA was stronger than that with dsDNA, which was consistent with the statement that HJNO binding with ssDNA was stronger than that with dsDNA. Compared with dsDNA, HJNO occupied more BLM^*642–1290*^ helicase binding sites to ssDNA when binding with it, thus exerting more intensive inhibiting on BLM^*642–1290*^ helicase binding to ssDNA. BLM helicase unzips the double strands towards 3′-5′ by binding with one of the partly unwinding strand [39]. Therefore, the strong HJNO inhibiting BLM^*642–1290*^ helicase binding with ssDNA promotes itself suppressing the DNA unwinding of BLM^*642–1290*^ helicase.

BLM helicase hydrolyzes ATP to release energy for unwinding DNA by its ATPase [40], thus we have detected the effect of HJNO on the ATPase of BLM^*642–1290*^ helicase. The results showed that HJNO had a certain suppression on the ATPase of BLM^*642–1290*^ helicase. Since the ATPase of BLM helicase depends on its DNA binding capacity [41], HJNO inhibiting the ATPase of BLM^*642–1290*^ helicase is related to its suppression on the DNA binding of BLM^*642–1290*^ helicase.

We further detected the effect of HJNO on the ultraviolet spectrum of BLM^*642–1290*^ helicase. The results showed that HJNO bound to BLM^*642–1290*^ helicase and changed its conformation. Theoretically, HJNO inhibited BLM^*642–1290*^ helicase by changing its conformation, however, it had a significant impact on changing BLM^*642–1290*^ helicase conformation when its concentration was 0.1 μmol/L, which was dramatically different from suppressive concentration range of BLM^*642–1290*^ helicase. The reason might be that HJNO could not inhibit BLM^*642–1290*^ helicase when its concentration was low, though a certain change of BLM^*642–1290*^ helicase conformation had occurred. When HJNO concentration was high, it could inhibit DNA binding of BLM^*642–1290*^ helicase by changing its conformation, by it inhibiting ATPase and DNA unwinding.

### The suppression of HJNO on MDA-MB-435 breast cancer cells expansion

We further confirmed the inhibitory effect of HJNO on tumor growth in MDA-MB-435 breast cancer cell culture a concentration-dependent manner. The results from colony forming assay found that HJNO also had a strong inhibitory effect on the colony formation of MDA-MB-435 breast cancer cells.

The mRNA and protein expression of BLM helicase in MDA-MB-435 breast cancer cells with HJNO treatment for 24 h were examined by RT-PCR and ELISA, respectively. And the results displayed an increasing pattern of them, which might contribute to HJNO inhibiting the DNA binding, ATPase and DNA unwinding of BLM helicase. Therefore, in order to resist HJNO soppressing BLM helicase, the mRNA and protein levels of BLM helicase in the MDA-MB-435 breast cancer cells increased through feedback when treated with HJNO. This also suggested that HJNO inhibited MDA-MB-435 breast cancer cells expansion by suppressing BLM helicase.

## Conclusion

Taken together, we screened out the potential anticancer small molecule HJNO by targeting BLM^*642–1290*^ helicase and further displayed its suppression on MDA-MB-435 breast cancer cells expansion. This was at least partly associated with HJNO suppressing BLM helicase. Therefore, our study provided some valuable clues for the study of HJNO in the living body and developing HJNO as an anticancer drug.

## Data Availability

All data and materials are available without restriction. Researchers can obtain data by contacting the corresponding authors.

## Abbreviations

BLM: Bloom syndrome helicase
BS: Bloom syndrome
EMSA: Electrophoretic mobility shift assay
HJNO: A tetrandrine derivative
HRDC: Helicase-ribonuclease D-C terminal domain
MDA-MB-435: MDA-MB-435 human breast cancer cell line
MMC: Mitomycin
MTT: Methyl thiazolyl tetrazolium
PBS: Phosphate Buffer solution
RecQ: RecQ helicase
RecQ-Ct: RecQ helicase C terminal
RT-PCR: Reverse transcription-polymerase chain reaction
RTS: Rothmund-Thomson syndrome
WRN: Werner syndrome helicase
WS: Werner syndrome
